# Orthotopic Ferret Tracheal Transplantation Using a Recellularized Bioengineered Graft Produces Functional Epithelia

**DOI:** 10.3390/bioengineering10070777

**Published:** 2023-06-29

**Authors:** Albert C. Pai, Anthony M. Swatek, Thomas J. Lynch, Bethany A. Ahlers, Vitaly Ievlev, John F. Engelhardt, Kalpaj R. Parekh

**Affiliations:** 1 Department of Cardiothoracic Surgery, University of Iowa Hospitals and Clinics, Iowa City, IA 52242, USA; albert-pai@uiowa.edu (A.C.P.); anthony-swatek@uiowa.edu (A.M.S.); 2 Department of Anatomy and Cell Biology, University of Iowa, Iowa City, IA 52242, USA; thomas-lynch@uiowa.edu (T.J.L.); bethany-ahlers@uiowa.edu (B.A.A.); vitaly-ievlev@uiowa.edu (V.I.); john-engelhardt@uiowa.edu (J.F.E.)

**Keywords:** tracheal transplant, tracheal revascularization, bioreactor, airway stem cells, ferret

## Abstract

Tracheal grafts may be necessary to bridge long-segment defects after curative resection for airway obstructions. Bioengineered grafts have emerged as an appealing option, given the possibilities of altering the histologic and cellular profile of the conduit. We previously designed a bioreactor capable of luminally decellularizing and recellularizing a ferret trachea with surface airway epithelia (SAE) basal cells (BCs), and we sought to assess the fate of these grafts when transplanted in an orthotopic fashion. As adjuncts to the procedure, we investigated the use of a vascular endothelial growth factor (VEGF)-laden hydrogel and of immunosuppression (IS) in graft revascularization and viability. IS was shown to limit early graft revascularization, but this effect could be counteracted with VEGF supplementation. Submucosal gland (SMG) loss was shown to be inevitable regardless of the revascularization strategy. Lastly, the bioengineered tracheas survived one month after transplant with differentiation of our implanted BCs that then transitioned into a recipient-derived functional epithelium. The work presented in this manuscript has important implications for future cellular and regenerative therapies.

## 1. Introduction

Malignant and benign tracheal obstructions are pathologic lesions where curative resections may generate a considerable defect. Removal of segments >50% of the total tracheal length in adults, or >30% in pediatric patients, will place undue tension on primary anastomoses and risk dehiscence of the repair. For this reason, there is a significant effort to identify an appropriate conduit to bridge the resulting gap. Several options have been explored, including autologous tissue composites, aortic allografts, synthetic prostheses, cadaveric tracheal transplantation, and bioengineered tracheas [1,2]. This latter category has introduced a discipline of stem cell biology wherein cadaveric tracheas can be decellularized and subsequently recellularized, ideally with autologous stem cells, such that a functional graft can be orthotopically transplanted with the recipient’s major histocompatibility complex (MHC) profile which theoretically limits organ rejection [3,4]. In a recent publication, our group introduced a novel bioreactor capable of partially decellularizing the luminal compartment of a ferret trachea and of supporting implanted allogeneic surface airway epithelial (SAE) basal cells (BCs). Furthermore, we demonstrated that establishing an air-liquid interface within the bioreactor promoted early differentiation of the SAE BCs [5]. In this article, we orthotopically transplanted these bioengineered tracheas and tracked the contribution of fluorescent airway stem cells. However, we had to address the issues of graft revascularization and immunosuppression to ensure a successful outcome for tracheal transplantation.

Tracheal transplantation is not a new concept and has been clinically attempted since the 1970s with limited success and significant morbidities related to graft ischemia [6,7]. The reason allotransplantation remains an exceptional challenge is due to the technical difficulty of revascularizing the organ. The trachea derives its intercartilaginous and mucosal blood supply from microvascular collateral branches provided by the inferior thyroid and bronchial arteries [8]. The absence of a vascularized pedicle to restore blood supply upon orthotopic transplantation leads to graft ischemia and eventual necrosis. While we do recognize the recent success of Genden et al. and their team’s long-segment transplant with microsurgical reanastomoses [9], tracheal revascularization has historically occurred via alternative means. Klepetko et al. first documented graft revascularization by wrapping a donor trachea in the recipient omentum [10]. Delaere et al. reported a case series of six patients who had cadaveric tracheas implanted in the forearms over two months to establish collaterals from a radial artery pedicle [11]. The ultimate lesson is that revascularization of the trachea occurs through inosculation—the direct anastomoses between the capillary buds of recipient blood to the adventitial blood vessels of the donor organ [2]. To promote this process of microvascular angiogenesis, localized supplementation of growth factors has been used in animal models, including basic fibroblast growth factor (b-FGF) and vascular endothelial growth factor (VEGF) [12,13,14,15]. Moreover, these studies suggested that treated allografts had accelerated and improved re-epithelialization [15,16,17], which is subsequently beneficial for graft survival and preventing obliterative lesions [18,19].

Another challenging aspect of airway transplantation is the perioperative use of immunosuppression (IS) to reduce the risk of graft rejection. Adverse effects of IS in this context include impaired bronchial healing after lung transplantation [20], and long-term use is associated with susceptibility to opportunistic infections, metabolic derangements, and cancers. Counterintuitively, studies also demonstrate improved tracheal revascularization and epithelialization with cyclosporine and methylprednisolone administration [21]. Fortunately, long-term IS use in tracheal transplants does not appear to be necessary, as Delaere et al. demonstrated that IS could be withdrawn after neovascularization and re-epithelialization of the donor trachea with recipient mucosa [11,22]. Identifying the exact effect of IS on early tracheal transplants is critical in predicting the longitudinal outcomes of bioengineered tracheas.

In this article, we investigate the issues presented above by performing orthotopic allotransplants with wild-type (WT) and bioengineered tracheas in sable ferrets. Revascularization and overall graft health, as assessed by submucosal glands (SMGs), epithelial presence, and airway patency, are challenged with VEGF supplementation and IS combinations. The fate of Tomato-fluorescent SAE BCs within transplanted bioengineered tracheas are tracked over the course of 31 days.

## 2. Materials and Methods

### 2.1. Animals

All animal experimentation was performed in accordance with approved protocols by the Institutional Animal Care and Use Committees of the University of Iowa. Wild-type (WT) adult sable ferrets were obtained from Marshall Farms (Rose, NY, USA). A dual-fluorescent Cre-reporter ferret was engineered by Crispr/Cas9-mediated homology-independent insertion, as described by Yu et al. [23]. Animals were housed in a ferret cage at the Animal Care Facility (Iowa City, IA, USA) with 12 h on/12 h off light exposure. Food and water were available ad libitum. The mean weight of the female and male ferrets was 0.735 kg and 1.565 kg, respectively. The mean age of all ferrets was 12.4 months.

### 2.2. Tissue Processing and Cell Isolation

Epithelial cells from ferret tracheas were isolated using a sequential enzymatic protocol as previously described [24]. In brief, dual-fluorescent ferrets were euthanized with an overdose of Euthasol^®^ (pentobarbital sodium and phenytoin sodium) and then topically sterilized with 70% ethanol. Whole-length tracheas were collected and decontaminated for 24 h in a solution of Minimum Essential Media (MEM) (ThermoFisher Scientific, Grand Island, NY, USA), imipenem/cilastatin (50 µg/mL), and 1% penicillin/streptomycin at 4 °C. Surface airway epithelium (SAE) were enzymatically isolated by digesting tissue in 5 mg/mL of Pronase (Roche, Indianapolis, IN, USA, CAS 9036-06-0) in F12 media for 60 min. Cells were then pelleted and cultured on 804G-conditioned plates with Pneumacult Ex-Plus (Stemcell Technologies, Vancouver, BC, CA, Cat#05008). Cells were cultured to passage 2 at the time of graft recellularization.

Ferret tracheas used for bioreactor recellularization and orthotopic transplantation were obtained from female WT tracheas. Under the same sterile conditions as described above, whole tracheas were procured. Adventitial tissue was sharply dissected from the trachea and subsequently stored in a decontamination solution prior to decellularization.

### 2.3. Bioreactor Decellularization and Recellularization

As described in our previous publication [5], female WT tracheas were processed as grafts for orthotopic tracheal transplantation. In brief, tracheas were trimmed to 5 cm in length and attached to the bioreactor. A sequential decellularization protocol of 1X DPBS, 0.25% *w*/*v* sodium dodecyl sulfate (SDS) in deionized water (diH_2_O), and then 1% *v*/*v* Triton X-100 at 37 °C maintained cartilage viability and de-epithelialized the lumen. Tracheas were thoroughly washed with 1X DPBS for 48 h at 4 °C prior to recellularization.

Processed tracheas were then trimmed to 3 cm and reattached to the bioreactor for recellularization. Tomato fluorescent ferret BCs were expanded to 80–90% confluence and collected with Accutase cell detachment solution (StemCell Technologies, Vancouver, BC, Canada, Cat#07920). The cell pellet was resuspended in 350 µL of Pneumacult Ex-Plus and injected as a bolus into the lumen of the trachea. The recellularization circuit was established, and expansion media was perfused for two days with regular graft washing.

### 2.4. Vascular Endothelial Growth Factor Hydrogel Synthesis

A genipin-crosslinked gelatin/agar hydrogel laden with vascular endothelial growth factor (VEGF) was synthesized as described by Gnavi et al. [25]. In brief, to produce five milliliters of hydrogel: 20 mg of agar (Sigma-Aldrich, Mannheim, Germany, CAS 9002-18-0) was dissolved in 5 mL of 1X DPBS at 90 °C for 60 min. The temperature was then reduced to 50 °C, and 80 mg of gelatin (Sigma-Aldrich, Mannheim, Germany, CAS 9000-70-8) was mixed in for 30 min. The temperature was then reduced to 37 °C and 2.5 µL of recombinant human VEGF (R&D Systems, Minneapolis, MN, USA, 293-VE-010) (100 µg/mL) was pipetted into the hydrogel for a final concentration of 50 ng/µL. In total, 1 mL syringe of aliquots of the hydrogel was sterilely prepared for tracheal transplantation and kept at 4 °C until use. All aliquots were used within 24 h of preparation.

### 2.5. Wild-Type-to-Wild-Type Tracheal Transplantation—Allogeneic Revascularization Study

Sixteen adult sable ferrets underwent allogeneic orthotopic tracheal transplantation. There was no intersex crossover of donors and recipients due to size mismatching. Male ferrets received tracheas from male ferrets of similar weight and age, and the same principle applied to females. The animals were randomly assigned into four treatment groups (n = 4 in each group; 2 females and 2 males) depending on whether immunosuppression (IS) was administered and whether they received a VEGF-hydrogel: IS+/VEGF+, IS−/VEGF+, IS+/VEGF−, and IS−/VEGF−.

Donor ferrets were induced with an interscapular injection of ketamine/xylazine (8 mg/kg and 4 mg/kg, respectively), and anesthesia was maintained with 1.5–5% isoflurane via single-lumen endotracheal intubation. Ferrets were placed supine and were sterilely prepped with Iodine and 70% ethanol from the lower jaw to the abdomen. A midline incision was made from the neck to the xiphoid process, and a median sternotomy was performed. The cranial and caudal vena cava were ligated and divided. The descending aorta was clamped, and 50 mL of Perfadex^®^ was flushed by gravity through a right ventriculotomy to purge the trachea of blood. Whole tracheas were then procured and devascularized by sharply removing the adventitia from the airway. Tracheas were stored in 1X DPBS at 37 °C for 30–60 min until transplantation, as described below.

Recipient ferrets were induced with anesthesia and intubated as above. The neck was prepped from the jaw to the sternal notch and draped in a sterile fashion. A longitudinal cervicotomy was performed, and the strap muscles were elevated. A segment of the trachea containing ten tracheal rings was then sharply excised. To ensure uninterrupted oxygenation, cross-table ventilation was applied by withdrawing the oral endotracheal tube (ETT) and placing a sterile ETT through the distal trachea from the surgical field. The donor trachea was also trimmed to ten tracheal rings in length and anastomosed in an orthotopic position with continuous 5-0 Prolene. There was no tension on the anastomoses, and the repair was buttressed with sternohyoid muscle. Animals in the VEGF group had 1 mL of hydrogel spread over the graft. Animals in the immunosuppression group were given daily cyclosporine (2 mg/kg), azathioprine (2 mg/kg), and methylprednisolone (2 mg/kg) for 7 days. Animals were monitored with bronchoscopy and computed tomography (CT) scan prior to sacrifice on a postoperative day (POD) 7.

### 2.6. Tracheal Transplantation—Recellularized Graft Study

**Figure 1:**
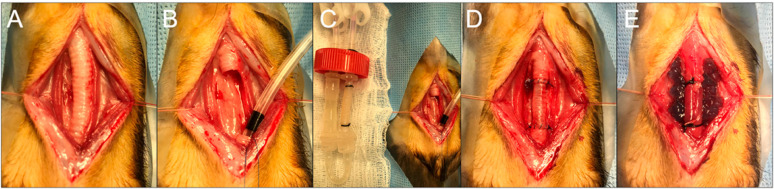
Orthotopic Tracheal Transplantation of the Bioengineered Trachea. (**A**) Longitudinal incision to expose the cervical incision. (**B**) A 10-ring segment is resected, and cross-table ventilation is resumed by inserting a sterile endotracheal tube through the distal tracheal segment. (**C**) A side-by-side comparison of the bioengineered trachea still attached to the bioreactor. (**D**) Orthotopic tracheal transplantation with 5-0 Prolene. (**E**) Sternohyoid muscle buttressing and hydrogel deposition.

Similar to above, eight recipient female ferrets were induced with anesthesia, and a ten-ring segment of the cervical trachea was excised. Bioreactor grafts on the second day of recellularization were trimmed to the same size and anastomosed in the same fashion as above. All animals had immunosuppression and VEGF hydrogel deposition. Four animals were sacrificed at 9 days, and the remainder (n = 4) were sacrificed at 31 days (Figure 1).

### 2.7. Bronchoscopy and Imaging

The 7 days WT-WT and 9 days bioengineered transplant cohorts had their anastomoses inspected on the day of sacrifice with a pediatric bronchoscope. The 31-day cohort had weekly bronchoscopies. CT imaging was also performed on the same schedule to evaluate for anastomotic dehiscence, graft viability, and luminal patency.

### 2.8. Tracheal Revascularization and Evaluation

Tracheal revascularization was assessed by intra-aortic injection of fluorescein isothiocyanate (FITC)-dextran (molecular weight 59,000–77,000) (Sigma-Aldrich, CAS 60842-46-8) at the time of sacrifice of the allogeneic tracheal transplants (not including the recellularized grafts; n = 16). A total of 80 mg/kg of FITC-dextran was dissolved in 10 mL of 1X DPBS. The cranial and caudal vena cava were ligated and divided, and the descending aorta was clamped. Ten milliliters of FITC-dextran were then injected into the ascending aorta to perfuse the tracheal vasculature. The tracheas were procured and analyzed under a Leica dissecting scope. Continuous variables were expressed as mean ± SD. The comparison groups were analyzed for significance (*p* < 0.05) with ANOVA for three or more groups and a two-tailed t-test for comparison between two treatment groups.

### 2.9. Tissue Analysis and Histology

All harvested tissue post-transplant was fixed in 4% paraformaldehyde (PFA) for 24 h at 4 °C and then trimmed to fit a cassette for OCT (Tissue-Tek O.C.T., Sakura, Torrance, CA, USA) embedding in liquid N_2_. Tissues were sectioned into 10 µm slices and fixed to slides in 4% PFA for 45 min. Antigen retrieval was performed by sodium citrate buffer (pH 6.0) submersion overnight at 55 °C. Blocking and permeabilization were conducted with donkey serum and 10% Triton X-100. Primary and secondary antibodies were sequentially incubated for 24 h at 4 °C. Slides were then imaged by Zeiss 880 confocal microscopy (Zeiss, Oberkochen, Germany). The comparison groups were analyzed for significance (*p* < 0.05) with ANOVA for three or more groups and a two-tailed t-test for comparison between two treatment groups.

## 3. Results

### 3.1. Allogeneic Transplant—Survival and Tracheal Patency

Sixteen WT animals were divided into four treatment groups depending on whether IS was administered and whether they received a VEGF-hydrogel: IS+/VEGF+, IS−/VEGF+, IS+/VEGF−, IS−/VEGF−. Two females and two males were included in each group. All animals survived for 7 days and underwent a CT scan and bronchoscopic exam prior to sacrifice. The oral intake on the POD1 was minimal, but their appetites returned to normal on POD2.

**Figure 2:**
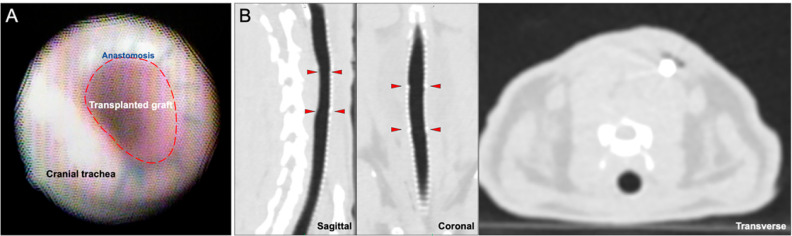
Bronchoscopic and Radiographic Surveillance at 7 Days Post-transplant. (**A**) Bronchoscopy shows a patent tracheal graft without evidence of necrosis. (**B**) Sagittal, coronal, and transverse views show intact tracheal anastomosis without stenosis or extraluminal air suggestive of dehiscence. The red arrowheads indicate the proximal and distal anastomotic boundaries.

Bronchoscopic examination in all tracheas showed normal, pink mucosa without patches of ischemia or necrosis. There was scattered hyperemia that was not attributable to any one treatment group. No suture line dehiscence was observed, and the pediatric bronchoscope was able to traverse both the proximal and distal anastomoses without difficulty (Figure 2A). CT showed overall luminal patency without extra-tracheal air (Figure 2B).

### 3.2. Allogeneic Transplant—Revascularization

**Figure 3:**
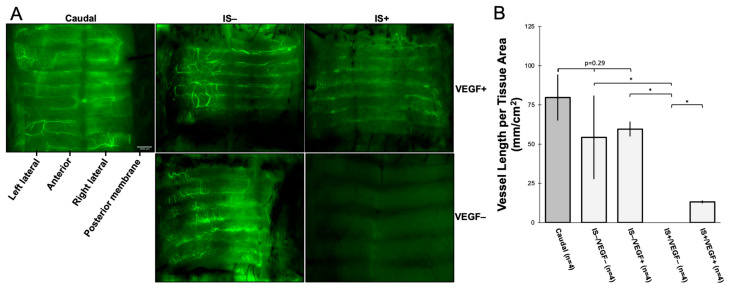
Revascularization of Tracheal Grafts. (**A**) The caudal trachea has normal-pattern vascularity. Untreated tracheas (IS−/VEGF−) are revascularized in a similar pattern with predominance in the left and right lateral regions. IS−/VEGF+ tracheas are also diffusely revascularized. Tracheas with dual treatment (IS+/VEGF+) are revascularized, but the vessel size is finer and more diffuse. Tracheas treated with only immunosuppression (IS+/VEGF−) show no revascularization at 7 days. Scale bar = 1000 µm. (**B**) The cumulative vessel length of vessels measuring at least 50 µm in diameter showed no significant difference between normal trachea vs. untreated trachea vs. VEGF+ only trachea (*p* = 0.29). There was significantly less revascularization in the IS+ only when compared with all other treatment groups, as denoted by an asterisk (*; *p* < 0.05). The dual-treatment trachea was significantly more revascularized than IS+ only tracheas, but the vessels are predominantly <50 µm wide.

All WT-WT animals were injected with intra-aortic FITC-dextran at the time of sacrifice. The tracheas were imaged with a GFP filter under a dissection microscope to visualize the tracheal vasculature. The results were compared to the vasculature of the caudal trachea in an otherwise normal WT ferret that had not undergone surgery. The cumulative length of patent vessels measuring ≥50 µm wide was estimated using FIJI software. This vascular dimension was arbitrarily selected for ease of identification and digital measurement. In ferrets who did not receive IS, the vessel length per tissue area (mm/cm^2^) was not significantly different compared to non-surgical trachea: normal trachea 79.74 ± 14.62 vs. IS−/VEGF− 54.33 ± 26.67 vs. IS−/VEGF+ 59.59 ± 4.75 (*p* = 0.29). In ferrets that received only IS, no vessels ≥50 µm wide were observed at 7 days post-transplant. Ferrets with IS and VEGF hydrogel supplementation demonstrated revascularization, but the number of vessels ≥50 µm was significantly reduced compared to the normal trachea or IS− tracheas: IS+/VEGF+ 13.22 ± 0.94 (*p* < 0.02) (Figure 3A,B).

**Figure 4:**
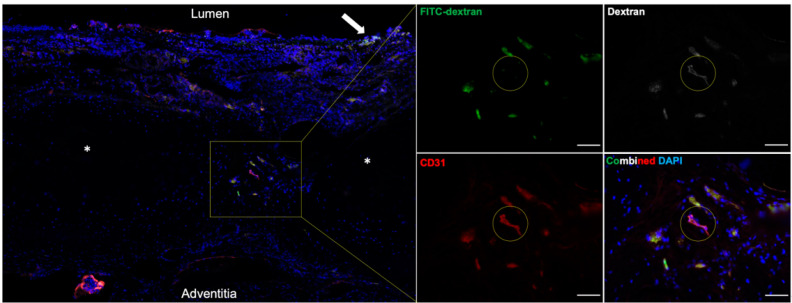
Immunohistology of Neovascularization within the Transplanted Trachea. The larger image depicts a cross-section of an immunosuppression-positive/VEGF-positive trachea. The inset focuses on a cluster of CD31-positive (red) blood vessels with FITC-positive staining (green). These demonstrate neovascularized blood vessels within the trachea. CD31-positive/FITC-negative regions, as shown by the yellow circle, represent endogenous blood vessels that have not been revascularized or are possibly thrombosed. Dextran (white) is also stained to serve as a positive control, but its presence in FITC-negative areas suggests autofluorescence. Asterisks (*) denote cartilage rings. The white arrow shows contamination of FITC-dextran on the luminal surface of the trachea during organ procurement. Scale bar = 50 µm.

Immunofluorescence staining for CD31 (endothelial cells), which identifies the intimal layer of blood vessels, and FITC-dextran was performed to obtain a ratio of perfused vasculature:total vasculature within the intercartilaginous spaces. Some sections, as shown in Figure 4, demonstrate FITC-absent blood vessels, which suggests previous endogenous blood vessels have not been revascularized at this time point. Due to the variable distribution of blood vessels—demonstrated in Figure 3A—within the transplanted trachea and autofluorescence of fixed samples, cross-sections demonstrating a meaningful difference between treatment groups could not be investigated. Additionally, FITC-dextran notably contaminated the trachea samples during procurement if uncontrolled exsanguination occurred, which resulted in FITC-positive regions that were CD31-negative. An example of this can be seen on the luminal surface of Figure 4 (arrow).

### 3.3. Allogeneic Transplant—Airway Histology

**Figure 5:**
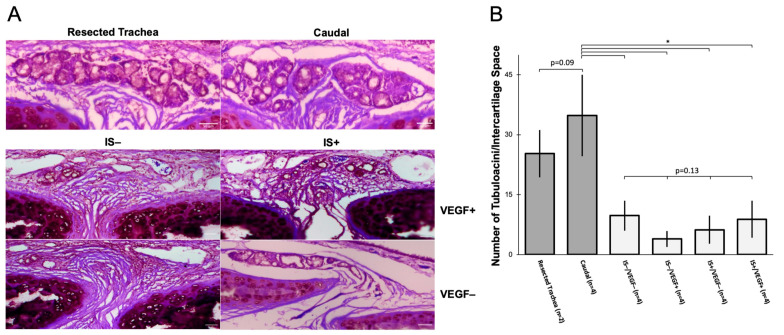
Submucosal Gland Tubuloacini Quantification (**A**) The resected 10-ring segment of the trachea and the caudal trachea have similar submucosal gland (SMG) morphology. In all treatment groups, there is notable atrophy of well-formed SMGs. Scale bar = 50 µm. (**B**) The number of tubuloacini per intercartilaginous space shows no difference in glandular structures between the resected and caudal trachea. However, all treatment groups had significantly fewer tubuloacini compared to caudal or resected trachea, as indicated by an asterisk (*; *p* < 0.05). There was no difference amongst treatment groups by ANOVA analysis.

Sections of WT-WT tracheal graft were stained in hematoxylin and eosin. The tubuloacini of the submucosal glands (SMGs) were quantified and compared against the tubuloacini of the otherwise normal trachea. Normal trachea in the region of transplantation averaged 25.27 ± 5.92 tubuloacini per intercartilaginous space. All treatment groups had significantly decreased amounts of tubuloacini compared to normal trachea, although there was no difference among the treatment groups: IS+/VEGF− 9.78 ± 3.77 vs. IS−/VEGF− 3.93 ± 1.98 vs. IS−/VEGF+ 6.21 ± 3.53 vs. IS+/VEGF+ 8.86 ± 4.66 (*p* = 0.13) (Figure 5A,B).

**Figure 6:**
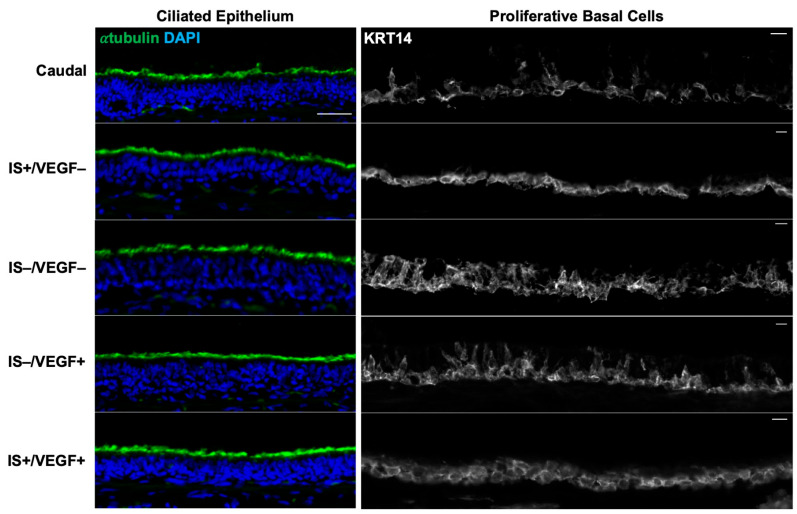
Immunohistology of Transplanted Tracheal Epithelium. α-tubulin (green) stains for luminal cilia and indicates the presence of ciliated epithelium. All treatment conditions maintained diffusely ciliated epithelium compared to that of the normal caudal trachea. Keratin 14 (white) is a marker of proliferative basal stem cells. There was no difference in the quantity of proliferative basal cells compared to the normal caudal trachea. DAPI indicates nuclei. Scale bar = 50 µm.

Immunofluorescent staining of the surface epithelium for α-tubulin and keratin (KRT) 14 showed preserved ciliated epithelium and basal cells across all treatment groups similar to unaffected caudal tracheas, respectively (Figure 6).

### 3.4. Recellularized Transplant

Tracheal transplants using Tomato-positive, keratin (KRT) 5-positive, and KRT14-positive SAE BC-engrafted tracheas were sacrificed at 9 days and 31 days post-transplant. All animals survived to their designated time points, and CT imaging and bronchoscopy showed no graft dehiscence and no graft patency.

**Figure 7:**
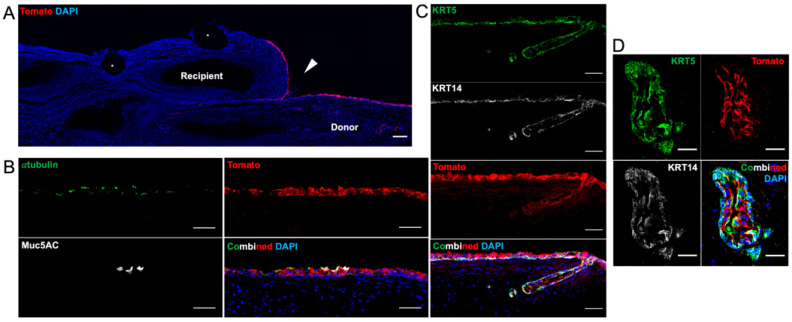
Immunofluorescence of Orthotopic Tracheal Transplant with Bioengineered Trachea—9 Days. (**A**) The transition point between the recipient and donor trachea shows engrafted Tomato-positive basal cells growing onto the recipient tracheal edge. The white triangle indicates the anastomotic line. Asterisks indicate suture. Scale bar = 100 µm. (**B**) Mucin5AC (white) and α-tubulin (green) staining indicates the presence of differentiated mucus-secreting goblet cells and ciliated cells, respectively. Note that all differentiated cells are derived from Tomato-positive cells. Scale bar = 50 µm. (**C**) Keratin (KRT) 5 (green) and KRT 14 (white) are airway basal cell markers indicating that the pseudostratified epithelium is generating differentiated cells and maintaining a basal stem cell layer. Scale bar = 50 µm. (**D**) A submucosal gland duct shows Tomato-positive basal cell colonization. There is a two-cell layer with a basal layer retaining KRT 5 and KRT 14. Scale bar = 25 µm.

Day 9 grafts all demonstrated survival of Tomato-positive engrafted cells with notable growth onto the recipient trachea (Figure 7A). Immunofluorescent staining for α-tubulin and Muc5AC showed Tomato-positive ciliated cells and goblet cells as well as a KRT5-positive and KRT14-positive basal layer (Figure 7B). All differentiated surface cells appeared to be derived from Tomato-positive cells. Engrafted cells are also seen in the submucosa forming a multilayer duct, but there was no organized structure representing the tubuloacini of a submucosal gland (Figure 7C,D).

**Figure 8:**
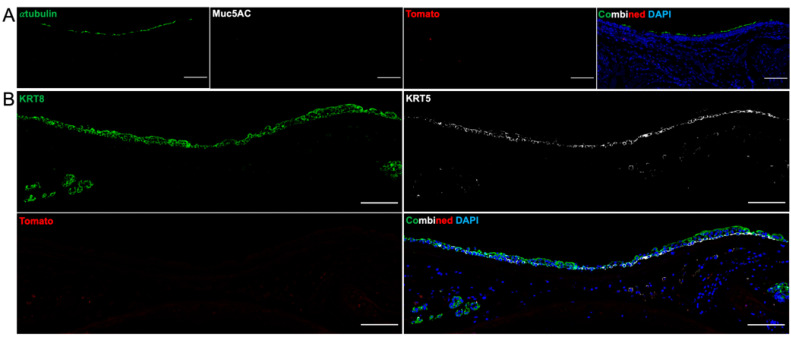
Immunofluorescence of Orthotopic Tracheal Transplant with Bioengineered Trachea—31 Days. (**A**) No cells derived from a Tomato-positive line are evident. There are scattered patches of α-tubulin-positive cells representing ciliated epithelium. These cells are Tomato-negative and are likely recipient-derived. There is no Muc5AC expression, suggesting that goblet cells are not differentiated. Scale bar = 100 µm. (**B**) Other patches of epithelium are Tomato-negative and multilayered. Cells are KRT 8-positive (green), which is a marker of cell commitment to luminal differentiation. KRT 5 (white) represents proliferative basal cells. Scale bar = 100 µm.

Day 31 grafts showed an epithelialized tracheal graft, but no cells in the SAE at 31 days were Tomato-positive. The epithelium alternated between α-tubulin-positive ciliated cells (Figure 8A) and immature, KRT 8-positive undifferentiated epithelium (Figure 8B). There were no organized SMG structures representing functional glands, but there was Hoescht staining suggesting nucleated cells populating the intercartilaginous spaces.

## 4. Discussion

Here, we present the results from a series of orthotopic tracheal transplants in ferrets that shed light on the effects of VEGF supplementation and IS on graft revascularization, as well as the performance of our bioengineered tracheas in situ. We selected ferrets as our animal model for several compelling reasons: (1) their trachea-to-body ratio is generous owing to their long necks, equating to a significant amount of accessible trachea for surgical work; (2) their airways are anatomically more analogous to humans (i.e., SMGs are developed throughout the entire cartilaginous airways, unlike rodents); (3) their histologic profile of chronic rejection in lung transplantation experiments are identical to those of humans; (4) the cost of maintenance per animal is considerably less than that of large animals; (5) the availability of a genetically engineered fluorescent ferret for cell lineage tracing; and (6) their airways are amenable to video bronchoscopy and cross-table ventilation [23,26,27,28]. Our bioengineered trachea was a luminally decellularized cadaveric trachea that was subsequently recellularized with allogeneic SAE BCs. We selected this conduit based on results from Aoki et al. [29], which demonstrated that cartilaginous ring viability preserved biomechanical integrity, and data from our group [5] further proved that this scaffold was capable of supporting and differentiating implanted stem cells.

However, to ensure the successful transplantation of these grafts, it was necessary to address the issues of organ revascularization and immunosuppression. Tracheal allotransplantation has historically been unfeasible because of the absence of a vascular pedicle for anastomosis. The trachea is dependent on numerous small blood vessels penetrating the trachea between the cartilage rings, and interruption of this blood supply leaves any detached segment with no mechanism of reperfusion, even if it were transplanted immediately. The majority of publications favor inosculation as the only means of tracheal revascularization. This can be accomplished by staged transplantation initiated by heterotopic implantation into the omentum or onto the forearm fascia to develop collaterals from larger arteries [10,11,22]. Other studies have shown the benefits of accelerated neoangiogenesis by supplementing transplants with b-FGF or VEGF [12,13,14,15]. In our orthotopic WT-WT allotransplants, we opted for simultaneous deposition of a biodegradable genipin-crosslinked gelatin/agar hydrogel laden with VEGF to optimize the chances of revascularization. This concept was initially designed for peripheral nerve growth and stimulation [25], but the straightforward preparation, the thixotropic and inert properties of the gel, and the sustained release of VEGF were important reasons to use this carrier in our experiments.

In our WT-WT allotransplants, use of the VEGF hydrogel was not associated with a significant increase in graft revascularization at 7 days (quantified by the summative length of vessels ≥ 50 µm per cm^2^ tissue) when compared against untreated transplanted tracheal tissue or non-surgical trachea. Conversely, there was a significant reduction of revascularization in transplants only treated with IS. In fact, there was a complete absence of visible blood vessels at 7 days. In animals that were treated with both VEGF and IS, there was notable revascularization, but this quantity of reperfused blood vessels was significantly less than in those animals without IS. These data suggest that inosculation occurs spontaneously within 7 days, and VEGF supplementation alone does not significantly impact the density of vascular reperfusion. However, IS does significantly inhibit the revascularization process within the first postoperative week, and this process is somewhat lessened with VEGF supplementation. This is not to say that IS results in no early revascularization; instead, there were no objectively visible vessels that were measured to be ≥50 µm in width. These results are clinically relevant since IS is a standard of care for allotransplantation. If IS is associated with delayed graft vascularization, this could manifest significant long-term ischemic consequences, including stenosis, rejection, or necrosis.

Histologic examination showed that all treatment groups maintained a normal-appearing ciliated epithelium. Compared to non-surgical tracheas, all groups displayed significant attrition of tubuloacini in the SMG. These structures are critical components of a functional airway as they participate in the innate immune system by secreting lysozyme and mucus, and an absence of SMGs in the tracheas results in increased bacterial infection [30]. Furthermore, myoepithelial cells (MECs), a reserve stem cell population residing in SMGs capable of glandular and surface epithelial repair after severe injury, are also lost. This places the conduit and recipient at risk of graft loss due to a reduced ability to respond to minor and major insults. The loss of SMGs in all treatment conditions also suggests that SMG loss is the consequence of ischemic injury, and early revascularization by 7 days is insufficient to rescue gland damage. Prior studies by Swatek et al. [28] suggested that denervation of a ferret lung lobe could also cause SMG alteration and experiments with longer timepoints will be required to elucidate the roles of ischemia and denervation in SMG loss.

Based on conclusions drawn about these proof-of-concept experiments, tracheal allotransplantation with the bioengineered tracheas was conducted with the VEGF hydrogel and IS. With the advent of a Tomato-positive Cre-reporter ferret [23], we were able to harvest and track the fate of KRT5-positive and KRT14-positive airway BCs implanted onto a decellularized graft. Nine days following the transplant, the engrafted tracheas showed retention of the fluorescent BCs on the luminal surface with limited migration onto the edges of the non-fluorescent recipient trachea. To our surprise, we also identified a pseudostratified epithelium with the scattered presence of α-tubulin-positive ciliated and MUC5AC-positive goblet cells in all the animals. To our knowledge, this is the first publication confirming that a bioengineered trachea is capable of hosting and differentiating the engrafted BCs after orthotopic transplantation. The SMGs, especially the tubuloacini, were notably absent throughout the tracheal grafts; however, superficial ducts were still present and colonized with a double layer of Tomato-positive cells. There was no lysozyme or mucin staining to indicate a functional SMG.

At 31 days post-transplant, all tracheal grafts remained patent, and there was a fully pseudostratified surface epithelium—alternating between ciliated epithelium and immature, KRT8-positive epithelium. However, there was no trace of Tomato fluorescence, suggesting that the engrafted cells were either completely rejected by the recipient immune system or were outcompeted by recipient epithelium migrating onto the graft. There were no formed SMGs nor immature placodes seen between the intercartilaginous spaces. This could represent that de novo SMG formation is unlikely to occur beyond embryogenesis or early postnatal life or that our choice of stem cell was not the ideal candidate for regenerating SMGs [31].

These data from the transplanted bioengineered tracheas carry major clinical implications. First, not only was our graft capable of supporting engrafted cells in vitro, but it was also able to maintain and differentiate the BCs into a functional epithelium in vivo by 9 days. This is a significant stepping stone in the field of tracheal engineering, as there has been controversy in identifying an ideal stem cell that is capable of regenerating ciliated and mucus-secreting epithelium. Second, there is an absence of SMGs within the graft, which equates to an absence of lysozymes, mucin, and MECs, essentially impairing the innate defense and repair mechanisms of the graft. SMG regeneration in vivo remains an elusive goal and perhaps could be accomplished if MECs had been engrafted, as we previously demonstrated in vitro [5]. Third, long-term grafts, by one-month post-transplant, had lost all traces of the engrafted epithelium. This could be a result of rejection due to MHC mismatch because the recipient epithelium had a competitive advantage and outgrew the implanted cells or both. Nevertheless, the end result was a patent trachea with a functional surface epithelium, most likely derived from the recipient. It is possible the engrafted Tomato-positive BCs are mere “placeholders” to prevent the formation of obliterative lesions [18,19] and that they prime the extracellular matrix with signals and growth factors until the recipient epithelium eventually takes over. If this is the case, our findings could shift the paradigm of tracheal engineering. Instead of selecting the ideal stem cell to engraft, the graft only requires a temporary epithelium until the recipient cells are able to assume dominance.

Our study is not without limitations. Chief among these is that the sample sizes of the WT-WT tracheal transplants were small and likely underpowered to detect a true difference between the revascularization and SMG atrophy. Nonetheless, there was a reportable difference, and larger sample sizes will be required to reinforce this finding. The WT-WT transplants with VEGF supplementation served as a proof of concept so that we could select a strategy for optimal revascularization. Secondly, we did not use autologous stem cells or reprogrammed induced-pluripotent BCs derived from the recipient to recellularize the bioengineered tracheas [32]. This would have been technically and financially burdensome to bronchoscopically harvest and expand the stem cells prior to engraftment. Instead, our experiment serves as a proof of concept that transplantation of the bioengineered tracheas and short-term maintenance of allogeneic stem cells is feasible. Another limitation is that we had no method of differentiating whether the cells that repopulated the 31-day transplants were derived from the recipient or from the residual cells of the WT donor trachea. We find it unlikely that an entire functional epithelium could be regenerated by MECs from an ischemic SMG within the donor graft, but a subset of Trop-2+ ductal cells [33] could potentially be a source of surface regeneration and warrants further investigation.

In summary, we present the findings of our orthotopic tracheal transplants in ferrets using bioengineered grafts. Important contributions from these experiments include the benefit of VEGF supplementation in the setting of concomitant IS administration for graft revascularization, anticipating the inevitable loss of SMGs during airway transplantation, and the viability of a bioengineered trachea maintaining an epithelium in vivo whether it be recipient- or donor-derived. Future experiments investigating the specifics of revascularization and using autologous stem cells and MECs can provide clinically translatable information on the long-term fate of engrafted cells and the feasibility of regenerating SMGs.

## Data Availability

Not applicable.
